# Direct Comparison of ^67^Ga-Scintigraphy and Fluorodeoxyglucose Positron Emission Tomography for the Evaluation of Cardiac Sarcoidosis

**DOI:** 10.1016/j.jacadv.2025.102336

**Published:** 2025-11-19

**Authors:** Shinichi Kurashima, Takeshi Kitai, Takeru Nabeta, Yoshihisa Naruse, Tatsunori Taniguchi, Chisato Miyakoshi, Yutaro Miyoshi, Hidekazu Tanaka, Ryota Morimoto, Yuichi Baba, Yutaka Furukawa, Yuya Matsue, Chisato Izumi

**Affiliations:** a Department of Heart Failure and Transplantation, National Cerebral and Cardiovascular Center, Osaka, Japan; b Department of Cardiovascular Medicine, Kitasato University School of Medicine, Sagamihara, Japan; c Division of Cardiology, Internal Medicine III, Hamamatsu University School of Medicine, Hamamatsu, Japan; d Department of Cardiovascular Medicine, Osaka University Graduate School of Medicine, Suita, Japan; e Center for Clinical Research and Innovation, Kobe City Medical Center General Hospital, Kobe, Japan; f Department of Cardiovascular Medicine, Kyoto University Graduate School of Medicine, Kyoto, Japan; g Division of Cardiovascular Medicine, Department of Internal Medicine, Kobe University Graduate School of Medicine, Kobe, Japan; h Department of Cardiology, Nagoya University Graduate School of Medicine, Nagoya, Japan; i Department of Cardiology and Geriatrics, Kochi Medical School, Kochi University, Nankoku, Japan; j Department of Cardiovascular Medicine, Kobe City Medical Center General Hospital, Kobe, Japan; k Department of Cardiovascular Biology and Medicine, Juntendo University Graduate School of Medicine, Tokyo, Japan

**Keywords:** cardiac sarcoidosis, diagnosis, fluorodeoxyglucose positron emission tomography, ^67^Ga scintigraphy

## Abstract

**Background:**

^67^Ga scintigraphy and 18F-fluorodeoxyglucose positron emission tomography (FDG-PET) are useful tools for diagnosing cardiac sarcoidosis (CS). However, its comparative diagnostic and prognostic values remain unclear.

**Objectives:**

This study aimed to assess the positivity rates, clinical characteristics, and prognosis of patients with CS who underwent both ^67^Ga scintigraphy and FDG-PET.

**Methods:**

We retrospectively studied 512 patients with CS who were enrolled in the ILLUMINATE-CS (ILLUstration of the Management and prognosIs of JapaNese PATiEnts with CS) registry. Among them, 101 patients who underwent both ^67^Ga scintigraphy and FDG-PET before immunosuppressive drug administration were analyzed. Myocardial segments showing uptake on ^67^Ga scintigraphy and FDG-PET/computed tomography were semiquantified using the American Heart Association’s 17-segment model.

**Results:**

The median age was 63 (56-70) years, 64 (63.4%) patients were female, and the median left ventricular ejection fraction was 48 (38-59)%. The patients were divided into 4 groups based on the findings of both examinations: 1) both-positive (n = 26, 25.7%); 2) only FDG-PET-positive (n = 72, 71.3%); 3) only ^67^Ga scintigraphy-positive (n = 0, 0%); and 4) both-negative (n = 3, 3.0%). Among the FDG-PET-positive patients, only 26 (26.5%) showed uptake on ^67^Ga scintigraphy. Conversely, 94.0% of the segments that showed uptake on ^67^Ga scintigraphy were also identified as positive segments on FDG-PET. During a median follow-up of 2.9 (1.4-5.3) years, the composite outcome of all-cause death, hospitalization for heart failure, and fatal ventricular arrhythmia showed no significant difference between patients in the both-positive group and those in the only FDG-PET positive group.

**Conclusions:**

^67^Ga scintigraphy demonstrates lower sensitivity for detecting myocardial inflammation in CS than FDG-PET and has limited prognostic significance.

Sarcoidosis is a multisystemic inflammatory disorder of unknown etiology characterized by noncaseating granulomas that primarily affecting the heart, lungs, eyes, and skin.^1^ Given that cardiac involvement, known as cardiac sarcoidosis (CS), significantly affects prognosis, accurate diagnosis and early treatment of CS are crucial.^2^, ^3^, ^4^, ^5^ Although clinically manifested CS was reported in only 5% of patients with sarcoidosis, cardiac lesions were observed in more than 25% of the autopsy cases.^6^^,^^7^ Moreover, recent advances in imaging techniques have further revealed that cardiac involvement is more common than previously thought. However, although histological confirmation is considered fundamental, the detection rate of endomyocardial biopsy is as low as 20% due to sampling errors.^8^^,^^9^ Therefore, clinical diagnostic criteria without histological proof in the myocardium exist to increase diagnostic sensitivity, and the keys for clinical diagnosis involve multimodality imaging.^3^^,^^10^ According to the Japanese Circulation Society guidelines, when histological confirmation is unproven, CS can be diagnosed when characteristic clinical or imaging features—such as wall motion abnormalities, ventricular aneurysm or thinning, complete atrioventricular block, or sustained ventricular tachycardia (VT)—are present, together with late gadolinium enhancement on cardiac magnetic resonance (CMR) as the primary imaging criterion, combined with evidence of myocardial inflammation detected by 18F-fluorodeoxyglucose (FDG) positron emission tomography (PET) or ^67^Ga scintigraphy.^10^ Historically, ^67^Ga scintigraphy has been used for the diagnosis of CS and the assessment of its activity^11^, ^12^, ^13^, ^14^, ^15^ and continues to be utilized globally. Recently, FDG-PET has been reported to have higher diagnostic sensitivity for detecting inflammation and is the preferred imaging modality for the diagnosis and management of CS.^16^^,^^17^ However, FDG-PET is not available at all facilities, and false positives are sometimes problematic because glucose metabolism may be elevated in other diseases such as ischemic heart disease, hypertrophic cardiomyopathy, and arrhythmogenic right ventricular cardiomyopathy.^18^^,^^19^ In contrast, ^67^Ga scintigraphy has low sensitivity but high specificity for inflammation.^16^ Thus, it remains controversial which imaging modality, ^67^Ga scintigraphy or FDG-PET, should be prioritized for investigating inflammation in CS.

To date, few studies have compared the positivity rates of FDG-PET and ^67^Ga scintigraphy, and none have examined their prognostic impact. Therefore, this study aimed to clarify the positivity rates, characteristics, and prognoses of patients who underwent both ^67^Ga scintigraphy and FDG-PET.

## Methods

### Study design and patient involvement

We utilized the ILLUMINATE-CS (ILLUstration of the Management and prognosIs of JapaNese PATiEnts with CS), a multicenter retrospective registry used to evaluate the clinical characteristics and outcomes of patients diagnosed with CS between 2001 and 2017. The study design and primary results of the ILLUMINATE-CS have been reported previously.^20^^,^^21^ In brief, the inclusion criterion of this study was a diagnosis of CS by the Heart Rhythm Society consensus statement or Japanese Circulation Society guidelines (Supplemental Table 1).^3^^,^^10^ Patients diagnosed before the development of the diagnostic criteria were verified to meet the current diagnostic criteria. All participants were informed about their participation in the study and that they were free to opt out of participation at any time. This study complied with the Declaration of Helsinki and the Japanese Ethical Guidelines for Medical and Health Research involving Human Subjects. The study protocol was approved by the ethics committee of each participating hospital (UMIN000034974).

**Figure 1:**
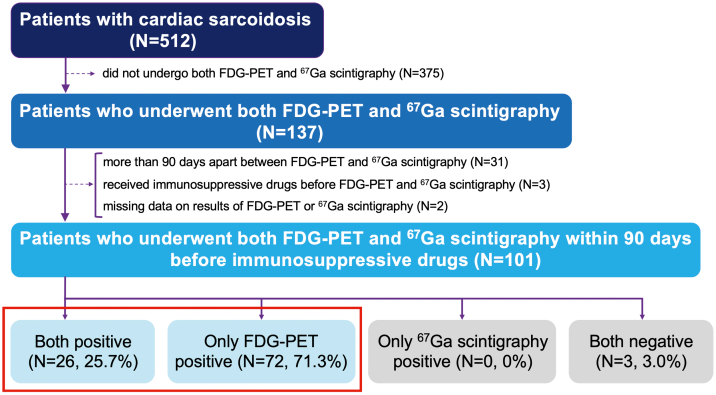
**Study Patient Flow** This flowchart shows the selection of 101 cardiac sarcoidosis patients who underwent both FDG-PET and ^67^Ga scintigraphy. FDG = 18F-fluorodeoxyglucose; PET = positron emission tomography.

Among the 512 patients from the ILLUMINATE-CS cohort, we selected 137 who underwent both FDG-PET and ^67^Ga scintigraphy. Patients whose scans were performed more than 90 days apart were excluded (n = 31). We also excluded patients who were administered immunosuppressive drugs before FDG-PET and ^67^Ga scintigraphy (n = 3) and those with missing data on the results of FDG-PET or ^67^Ga scintigraphy (n = 2). Consequently, 101 patients were included in this study (Figure 1).

### Imaging analysis

All imaging analyses were performed according to standardized protocols by experienced site investigators. For FDG-PET, participating institutions generally followed the recommendations of the Japanese Society of Nuclear Cardiology, which included a low-carbohydrate/high-fat diet on the day before imaging, followed by at least 12 hours of fasting to suppress physiological myocardial glucose uptake. FDG was administered intravenously at a dose of 111–259 MBq (2–5 MBq/kg) for 3D data collection, and 185–444 MBq (3–7 MBq/kg) for 2D data collection.^22^ For ^67^Ga scintigraphy, although institutional protocols varied, most sites performed planar and single-photon emission computed tomography imaging 48 to 72 hours after the intravenous administration of 74 to 111 MBq of ^67^Ga-citrate.^10^ The American Heart Association 17-segment model was used to evaluate the distribution and semiquantify the myocardial segments showing uptake on FDG-PET/computed tomography and ^67^Ga scintigraphy.^20^^,^^23^

### Follow-up and clinical events

Clinical follow-up data were collected from medical records, direct contact, or telephone interview with the referring physician at each hospital. The primary outcome measure in the ILLUMINATE-CS was a composite of all-cause death, hospitalization for heart failure, and fatal ventricular arrhythmic events (FVAEs). FVAEs include sudden cardiac death, ventricular fibrillation, sustained VT (>30 s), and appropriate implantable cardioverter-defibrillator therapy. Causes of death, including sudden cardiac death and heart failure hospitalization, were defined according to the definitions recently proposed by the Heart Failure Collaboratory and Academic Research Consortium.^24^ These outcomes were standardized across the ILLUMINATE-CS studies.

### Statistical methods

Categorical variables are represented as numbers (percentages) and were compared using the chi-square test or the Fisher exact test, as appropriate. Continuous variables were expressed as medians and IQRs. Depending on their distribution, which was assessed qualitatively through histograms and Q-Q plots, continuous variables were compared with an unpaired 2-tailed *t*-test when normally distributed or with the Wilcoxon rank sum test or Mann-Whitney *U* test when not normally distributed. Two-sided *P* < 0.05 was regarded as statistically significant. The Kaplan-Meier method was used to estimate the cumulative incidence of events, and differences were evaluated using the log-rank test. Statistical analyses were performed using JMP, version 18.0.0 (SAS Institute Inc).

## Results

### Patient characteristics

The median age was 63 years (IQR: 56–70), 64 patients (63.4%) were female, and the median left ventricular ejection fraction (LVEF) was 48% (38–59). CMR was performed in 54 of the 101 patients (53.5%), of whom 43 (79.6%) underwent CMR prior to FDG-PET or ^67^Ga scintigraphy. Late gadolinium enhancement was observed in 98.1% of these patients. The patients were divided into 4 groups based on the findings of both FDG-PET and ^67^Ga scintigraphy: 1) both-positive (n = 26, 25.7%); 2) only FDG-PET-positive (n = 72, 71.3%); 3) only ^67^Ga scintigraphy-positive (n = 0, 0%); and 4) both-negative (n = 3, 3.0%) (Figure 1). Given the absence of patients in the only ^67^Ga scintigraphy-positive group and the minimal number in the both-negative group, further analyses focused on comparing the both-positive and the only FDG-PET-positive groups. The characteristics of the patients in the both-positive group and the only FDG-PET-positive group are shown in Table 1. Patients in the only FDG-PET-positive group had a higher prevalence of LVEF <40% and septal thinning and were more frequently prescribed angiotensin-converting enzyme inhibitors or angiotensin II receptor blockers, beta-blockers, and amiodarone at baseline than those in the both-positive group. No significant differences were observed in the complication rates of sustained VT or ventricular fibrillation and complete atrioventricular block. No significant differences were observed in the proportion of patients who received corticosteroid therapy. The both-negative group consisted of 3 patients with clinical CS and systemic sarcoidosis (Supplemental Table 2).

**Table 1:** Baseline Characteristics of Patients

	Both-Positive (n = 26)	Only FDG-PET-Positive (n = 72)	*P* Value
Age, y	64 (57-67)	63 (56-71)	0.394
Female	21 (80.8%)	41 (56.9%)	**0.031**
NYHA functional class			0.653
I/II	22 (88.0%)	59 (84.3%)	
III/IV	3 (12.0%)	11 (15.7%)	
LVEF, %	56 (44-60)	48 (36-62)	0.058
LVEF >50%	15 (57.7%)	30 (42.9%)	0.196
LVEF <40%	3 (11.5%)	22 (31.4%)	**0.049**
Septal thinning	6 (24.0%)	33 (46.5%)	**0.049**
LV aneurysm	2 (8.3%)	10 (14.1%)	0.463
CMR	12 (46.2%)	39 (54.2%)	0.483
LGE-positive	12 (100%)	37 (97.4%)	0.570
Prior heart failure hospitalization	5 (20.8%)	16 (22.5%)	0.862
AV block	17 (68.0%)	35 (49.3%)	0.107
Sustained VT or VF	3 (13.0%)	14 (19.4%)	0.486
Nonsustained VT	3 (13.0%)	13 (18.6%)	0.542
Pacemaker implantation	12 (48.0%)	28 (39.4%)	0.455
CRTP	0 (0%)	0 (0%)	
ICD implantation	0 (0%)	8 (11.4%)	0.083
CRTD	1 (4.2%)	4 (5.7%)	0.771
Comorbidities			
Hypertension	3 (12.0%)	29 (41.4%)	**0.008**
Dyslipidemia	3 (13.0%)	13 (18.6%)	0.542
Diabetes mellitus	7 (28.0%)	21 (29.6%)	0.881
Atrial fibrillation	1 (4.4%)	7 (9.9%)	0.410
Coronary artery disease	0 (0%)	4 (5.6%)	0.235
Laboratory data			
BNP, pg/mL	119 (38-221)	151 (56-313)	0.601
Creatinine, mg/dL	0.75 (0.66-0.87)	0.79 (0.68-1.06)	0.240
ACE, U/L	16.7 (13.0-24.4)	15 (10.9-20.8)	0.076
sIL2R, U/mL	477 (337-746)	528 (372-790)	0.467
Medications			
ACEI or ARB	8 (30.8%)	44 (61.1%)	**0.008**
β-blocker	3 (12.0%)	37 (51.4%)	**<0.001**
Amiodarone	0 (0%)	11 (15.3%)	**0.038**
Corticosteroid therapy	26 (100%)	64 (88.9%)	0.076
Classification of biopsy-proven or biopsy-not-proven CS			
Biopsy-proven CS	15 (57.7%)	38 (52.8%)	0.666
From the heart (HRS definite)	4 (15.4%)	7 (9.7%)	0.433
From other organs (HRS probable)	11 (42.3%)	31 (43.1%)	0.947
Biopsy-not-proven CS (JCS only)	11 (42.3%)	34 (47.2%)	0.666
Classification of CS with systemic or isolated CS			
CS with systemic sarcoidosis	21 (80.8%)	54 (75.0%)	0.552
Isolated CS	5 (19.2%)	18 (25.0%)	0.552
Histological isolated CS	2 (7.7%)	2 (2.8%)	0.278
Clinical isolated CS	3 (11.5%)	16 (22.2%)	0.238

Values are n (%) or median (IQR).

ACE = angiotensin-converting enzyme; ACEI = angiotensin-converting enzyme inhibitor; ARB = angiotensin II receptor blocker; AV = atrioventricular; BNP = B-type natriuretic peptide; CMR = cardiac magnetic resonance; CRTD = cardiac resynchronization therapy defibrillator; CRTP = cardiac resynchronization therapy pacemaker; CS = cardiac sarcoidosis; FDG = 18F-fluorodeoxyglucose; HRS = Heart Rhythm Society; ICD = implantable cardioverter-defibrillator; JCS = Japanese Circulation Society; LGE = late gadolinium enhancement; LV = left ventricular; LVEF = left ventricular ejection fraction; PET = positron emission tomography; sIL2R = soluble interleukin-2 receptor; VF = ventricular fibrillation; VT = ventricular tachycardia.

### Imaging findings

**Figure 2:**
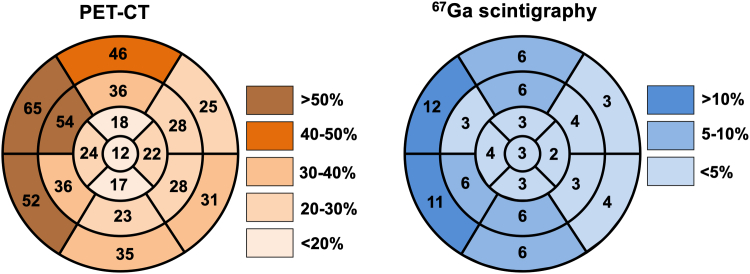
**The Difference in Positivity Rates Between FDG-PET and ^67^Ga Scintigraphy** FDG-PET was positive in 97.0% of patients, whereas ^67^Ga scintigraphy was positive in only 25.7%. CT = computed tomography; other abbreviations as in Figure 1.

**Figure 3:**
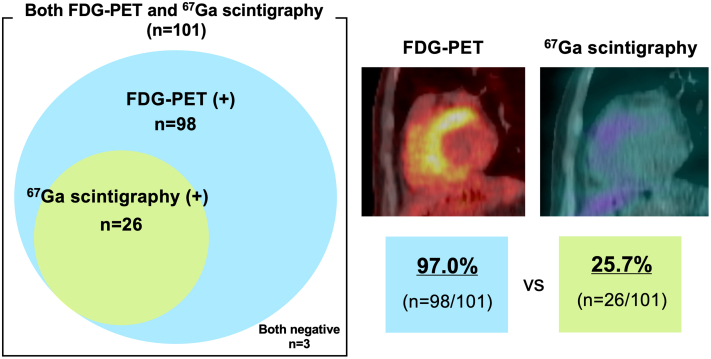
**Distributions of Uptake in FDG-PET and ^67^Ga Scintigraphy** FDG and ^67^Ga uptake were shown in 17 segments of the myocardium. Abbreviations as in Figure 1.

The median interval between FDG-PET and ^67^Ga scintigraphy was 19 (6–35) days. Among the 101 patients, FDG-PET was positive in 98 (97.0%), whereas ^67^Ga scintigraphy was positive in only 26 (25.7%) (Figure 2). All 26 patients with positive ^67^Ga scintigraphy findings also had positive FDG-PET findings. The distributions of uptake on FDG-PET and ^67^Ga scintigraphy are shown in Figure 3. Comparing the uptake segments of FDG-PET and ^67^Ga scintigraphy, the uptake segments on FDG-PET included 94.0% of the segments with uptake on ^67^Ga scintigraphy.

### Clinical outcomes

**Figure 4:**
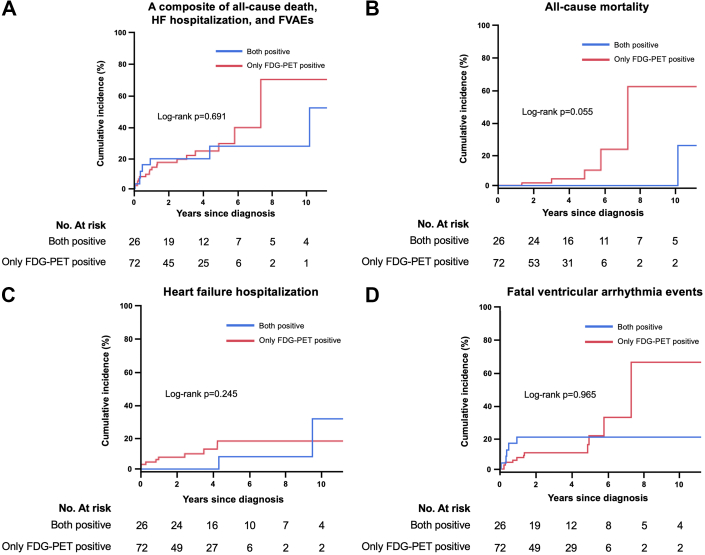
**Survival Curves Between the Both-Positive vs the Only FDG-PET-Positive Groups** (A) primary outcome measure, defined as a composite of all-cause death, heart failure hospitalization, and FVAEs; (B) all-cause mortality; (C) heart failure hospitalization; (D) FVAEs, defined as a composite of sudden cardiac death, VF, sustained VT and appropriate ICD therapy. FVAEs = fatal ventricular arrhythmic events; HF = heart failure; ICD = implantable cardioverter-defibrillator; VF = ventricular fibrillation; VT = ventricular tachycardia; other abbreviations as in Figure 1.

The incidences of adverse events are shown in Table 2. During a median follow-up of 2.9 (1.4–5.3) years, the risks of the primary outcome measure were comparable between patients in the only FDG-PET-positive group and those in the both-positive group (Figure 4A). The results were similar for all-cause death, heart failure hospitalization, and FVAEs (Figures 4B to 4D).

**Table 2:** Comparisons of Adverse Events

	Both Positive (n = 26)	Only FDG-PET Positive (n = 72)	*P* Value
Primary outcome measure^a^	7 (26.9%)	18 (25.0%)	0.847
All-cause death	1 (3.9%)	5 (6.9%)	0.572
Heart failure hospitalization	2 (7.7%)	8 (11.1%)	0.622
FVAEs^b^	5 (19.2%)	11 (15.3%)	0.640
Sudden cardiac death	0 (0%)	3 (4.2%)	0.290
Ventricular fibrillation	0 (0%)	1 (1.4%)	0.554
Sustained ventricular tachycardia	4 (15.4%)	6 (8.6%)	0.332
Appropriate ICD therapy	3 (15.0%)	5 (8.9%)	0.448
Nonsustained ventricular tachycardia	9 (39.1%)	15 (23.8%)	0.161
AV block	1 (3.9%)	4 (5.7%)	0.714
Device implantation	10 (38.5%)	12 (17.1%)	0.027

Values are n (%).

FVAEs = fatal ventricular arrhythmic events; other abbreviations as in Table 1.

a defined as a composite of all-cause death, heart failure hospitalization, and FVAEs.

b defined as a composite of sudden cardiac death, ventricular fibrillation, sustained ventricular tachycardia, and appropriate ICD therapy.

## Discussion

This study investigated the benefits of FDG-PET compared with those of ^67^Ga scintigraphy in terms of diagnostic sensitivity and prognostic implications (Central Illustration). The key findings of this study include the following: 1) many patients showed FDG uptake but not ^67^Ga uptake, and no patients were positive only for ^67^Ga scintigraphy; 2) in the both-positive group, the FDG uptake segments encompassed the majority of the segments showing ^67^Ga uptake; and 3) the prognoses were similar between the both-positive group and the only FDG-PET-positive group.

**Central Illustration:**
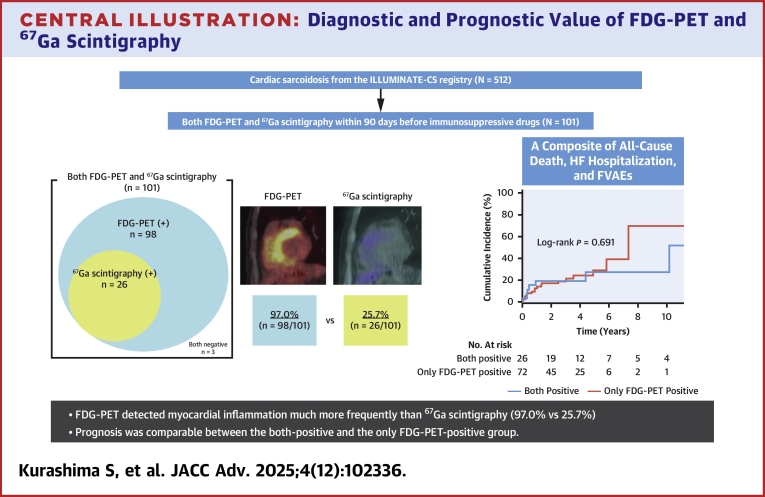
**Diagnostic and Prognostic Value of FDG-PET and ^67^Ga Scintigraphy** Among 512 patients with cardiac sarcoidosis enrolled in the ILLUMINATE-CS registry, 101 underwent both ^67^Ga scintigraphy and FDG-PET prior to initiation of immunosuppressive therapy. The Venn diagram shows positivity by modality: FDG-PET was positive in 97.0%, whereas ^67^Ga scintigraphy was positive in only 25.7%. All ^67^Ga-positive cases were also FDG-PET-positive and no patient was ^67^Ga-positive alone. The Kaplan-Meier curves show no significant difference in outcomes between the both-positive and the only FDG-PET-positive group (log-rank *P* = 0.691). ILLUMINATE-CS = ILLUstration of the Management and prognosIs of JapaNese PATiEnts with Cardiac Sarcoidosis; other abbreviations as in Figures 1 and 4.

The important clinical features of CS include the responsiveness to immunosuppressive therapy and the high occurrence of FVAEs, highlighting the need for accurate diagnosis and early treatment.^20^^,^^25^ Although histological confirmation remains fundamental for diagnosing sarcoidosis, it is challenging to obtain in all patients due to the low positivity rate of endomyocardial biopsy.^8^^,^^9^^,^^20^^,^^25^ Given these circumstances, the criteria for clinical diagnosis have been established to enhance the diagnostic rate.^3^^,^^10^ These findings should also be interpreted in light of the latest European Society of Cardiology consensus on the management of CS, which emphasizes multimodality imaging and individualized patient management, as well as the diagnostic criteria issued by the World Association of Sarcoidosis and Other Granulomatous Disorders, which remain a key reference for clinical and research settings.^26^^,^^27^ Although ^67^Ga scintigraphy has been used for many years for the diagnosis of sarcoidosis, its diagnostic utility compared to that of FDG-PET has recently been questioned.^28^ In this study, no patient was diagnosed based only on the ^67^Ga scintigraphy uptake, and only 26.5% of the FDG-PET-positive patients showed ^67^Ga scintigraphy uptake. This finding is consistent with previous reports that ^67^Ga scintigraphy has lower diagnostic sensitivity than FDG-PET in sarcoidosis.^17^^,^^29^^,^^30^ However, the study by Yamagishi et al was based on a very small cohort of 18 patients, which limited the strength of the evidence. Additionally, we found that 94.0% of the segments showing ^67^Ga uptake also showed FDG uptake in the both-positive group. If only ^67^Ga scintigraphy had been performed without FDG-PET, 19 (18.8%) patients would not have met the diagnostic criteria and would not have been diagnosed with CS. While several studies have shown a link between uptake on ^67^Ga scintigraphy and disease activity, these studies are limited in sample size, and the relationship between disease activity and long-term prognosis is unclear.^13^, ^14^, ^15^ On the other hand, clinical events such as death and VT are increased in patients with extensive myocardial uptake, right ventricular uptake, or perfusion-metabolism mismatch on FDG-PET.^31^, ^32^, ^33^, ^34^ Furthermore, FDG-PET plays a crucial role in assessing treatment response. Decreased uptake after treatment was associated with an improved LVEF and fewer cardiovascular events.^35^^,^^36^ Therefore, it may be helpful to repeat FDG-PET to develop effective treatment strategies.^37^^,^^38^ Although the results of the present study suggest that ^67^Ga scintigraphy alone is insufficient to evaluate disease activity and may lead to a worse prognosis due to inadequate treatment, a clinical dilemma still exists because many institutions worldwide can only perform ^67^Ga scintigraphy.

One reason for the lower diagnostic sensitivity of ^67^Ga scintigraphy might be its poorer spatial resolution compared to that of FDG-PET, leading to reduced detection of inflammation in smaller areas or those with lower intensity. Therefore, we hypothesized that patients with positive FDG-PET but negative ^67^Ga scintigraphy may have a better prognosis than those with both positive results. This assumption is based on the belief that the extent and degree of inflammation would be smaller and lower in patients in the only FDG-PET-positive group, possibly reflecting an early stage of disease. However, the prognosis was comparable between patients in the only FDG-PET-positive group and those in the both-positive group. Another assumption is that only FDG-PET-positive may reflect late disease stages. When inflammation is active, both tests are likely positive. In contrast, as inflammation progresses, tissue necrosis occurs in the myocardial tissue, and the degree of inflammation may decrease, resulting in only FDG-PET-positive, similar to the early disease stage. Indeed, the patients in the only FDG-PET-positive group had a higher prevalence of reduced LVEF and septal thinning. While the reason for the higher prevalence of hypertension remains unclear, the higher β-blocker use in the only FDG-PET-positive group may reflect the larger proportion of patients with LVEF <40% in this group. The higher rate of device implantation in the both-positive group, despite their better baseline LVEF and similar rates of ventricular arrhythmia and high-grade atrioventricular block, may reflect a treatment-selection bias.

Although the results of this study suggest that there is little advantage in performing ^67^Ga scintigraphy in CS, the distinct mechanisms of uptake between ^67^Ga scintigraphy and FDG-PET may enhance the diagnostic specificity for CS. Glucose is the primary energy source for the myocardium, and FDG uptake occurs physiologically in the myocardium via glucose transporter (GLUT) type 4. The mechanism of FDG uptake into inflammatory cells is similar to that in cancer cells, involving increased expression of GLUT1 and GLUT3 in the plasma membrane and activation of hexokinase.^39^ In the fasting state, glucose utilization mediated by GLUT4 is suppressed by increased levels of blood fatty acids. Consequently, prolonged fasting, switching to a low-carbohydrate/high-fat diet, and intravenous heparin can suppress physiological FDG uptake into the myocardium and reveal FDG uptake in the lesion. However, even with these interventions, physiological uptake can still be observed in up to 20% of patients.^40^ In addition, FDG uptake in the myocardium does not necessarily indicate inflammation. Because FDG is also taken up at sites of glucose hypermetabolism caused by chronic ischemia, it can be taken up in pathologies such as ischemic heart disease, hypertrophic cardiomyopathy, and severely impaired cardiac function, potentially leading to a false-positive diagnosis of CS.^18^^,^^19^^,^^41^

In addition to diagnostic performance, the radiation burden and resource considerations for each modality should be acknowledged. The typical effective dose for ^67^Ga scintigraphy is approximately 15 mSv, whereas that for FDG-PET is approximately 14 mSv^42^; however, these values vary depending on the patient weight, imaging protocols, and equipment. The availability, cost, and institutional resources may also influence the choice of imaging modality, particularly in facilities where FDG-PET is not accessible.

### Strengths and limitations

The strengths of the present study include the analysis of a large cohort of patients who underwent both FDG-PET and ^67^Ga scintigraphy, which provided insights into the long-term prognosis of these patients. However, this study has several limitations. First, this was a retrospective analysis with inherent limitations. Second, the preparation and acquisition protocols for both imaging modalities were determined by each participating institution and were not fully standardized across the registry, which may have introduced temporal and interinstitutional variability. In addition, the interpretations of the imaging were without central or blinded adjudication. Although standardized protocols and investigator training have been implemented to reduce intersite variability, the absence of a central review may have introduced heterogeneity in image interpretation. Third, we selected patients who underwent both tests within a relatively short period and the tests were not performed on the same day. Finally, none of the patients in our cohort exclusively demonstrated myocardial uptake on ^67^Ga scintigraphy. This finding may reflect limited statistical power rather than definitive evidence of the diagnostic inferiority of the modality. Despite these limitations, our study provides significant insights into the clinical management of CS.

### Clinical implications

The present findings reinforce the superior diagnostic sensitivity of FDG-PET over ^67^Ga scintigraphy for detecting CS. Continued reliance on ^67^Ga scintigraphy in settings where FDG-PET is unavailable may lead to underdiagnosis and potential delays in initiating appropriate treatment.

## Conclusions

In patients with CS, ^67^Ga scintigraphy demonstrates lower sensitivity for detecting myocardial inflammation than FDG-PET and has limited prognostic significance. These findings support FDG-PET as the preferred diagnostic modality, while reliance on ^67^Ga scintigraphy when PET is unavailable may lead to underdiagnosis.

Perspectives **COMPETENCY IN MEDICAL KNOWLEDGE:** In patients with CS who underwent both modalities, only 25.7% of the patients were positive for both modalities, and none of the patients were positive only for ^67^Ga scintigraphy. In the both-positive group, the segments identified with FDG uptake encompassed the majority of those also showing ^67^Ga uptake. Moreover, the prognosis was similar between the both-positive group and the only FDG-PET-positive group. ^67^Ga scintigraphy falls short in diagnostic precision and risk stratification for CS compared to FDG-PET. With its superior positivity rate and role in guiding clinical decisions, FDG-PET may be considered the preferred initial imaging modality for CS when available.

**TRANSLATIONAL OUTLOOK:** The findings challenge ^67^Ga scintigraphy’s diagnostic utility, advocating for FDG-PET as the primary imaging tool in CS. Prospective studies are needed to evaluate FDG-PET-guided treatment strategies and their impact on long-term outcomes.

## Funding support and author disclosures

Dr Hidehkazu Tanaka is a consultant for 10.13039/100004325AstraZeneca plc, Ono Pharmaceutical Company, Limited, 10.13039/100004319Pfizer Inc, and 10.13039/100004336Novartis International AG. Dr Yuya Masue has received an honorarium from Otsuka Pharmaceutical Co and 10.13039/100030831Novartis Japan. All other authors have reported that they have no relationships relevant to the contents of this paper to disclose. ILLUMINATE-CS was partially supported by the Novartis Pharma Research Grants.
