# Supplementary material for: Direct Comparison of 67Ga-Scintigraphy and Fluorodeoxyglucose Positron Emission Tomography for the Evaluation of Cardiac Sarcoidosis
Source: JACC Adv. 2025 Nov 19;4(12):102336. doi: 10.1016/j.jacadv.2025.102336 (PMC12670090; doi:10.1016/j.jacadv.2025.102336)
Supplement: Supplemental Tables 1 and 2 [file mmc1.pdf]

Supplemental Table 1. Comparison between HRS and JCS criteria

Heart Rhythm Society (HRS) criteria (2014)	Japanese Circulation Society (JCS) criteria (2016)
1. Histological diagnosis from myocardial tissue. CS is diagnosed in the presence of non-caseating granuloma on histological examination of myocardial tissue with no alternative cause identified (including negative organismal stains if applicable).	1. Histological diagnosis group (those with positive myocardial biopsy findings)<br>2. Clinical diagnosis group (those with negative myocardial biopsy)<br>1) Epithelioid granulomas are found in organs other than the heart, and clinical findings strongly suggestive of cardiac involvement are present.<br>2) The patient shows clinical findings strongly suggestive of pulmonary or ophthalmic sarcoidosis and at least 2 of the 5 characteristics laboratory** findings of CS and clinical findings are strongly suggestive of CS.
2. Clinical diagnosis from invasive and non-invasive studies. It is probable* that there is CS if:<br>a) There is a histological diagnosis of extra-cardiac sarcoidosis.<br>and	Criteria for cardiac involvement:<br>A) 2 or more of the 5 major criteria (a) to (e) are satisfied.<br>B) 1 of the 5 major criteria (a) to (e) and 2 or more of the 3 minor criteria (f) to (h) are satisfied.<br><br>Major criteria:

b) One or more of following is present:<br>• Steroid +/- immunosuppressant responsive cardiomyopathy or heart block.<br>• Unexplained reduced LVEF (<40%).<br>• Unexplained sustained (spontaneous or induced) VT.<br>• Mobitz type II 2nd degree heart block or 3rd degree heart block.<br>• Patchy uptake on dedicated cardiac PET (in a pattern consistent with CS).<br>• Late gadolinium enhancement on CMR (in a pattern consistent with CS).<br>• Positive gallium uptake (in a pattern consistent with CS)<br>and<br>c) Other causes for the cardiac	a) High-grade atrioventricular block or fatal ventricular arrhythmia (sustained VT or VF).<br>b) Basal thinning of the ventricular septum or abnormal ventricular wall anatomy (ventricular aneurysm, thinning of the middle or upper ventricular septum, regional ventricular wall thickening).<br>c) Left ventricular contractile dysfunction (LVEF <50%) or focal ventricular wall asynergy.<br>d) ^{67}Ga citrate scintigraphy or FDG-PET reveals abnormal accumulation in the heart.<br>e) Gadolinium-enhanced CMR reveals delayed contrast enhancement of the heart.<br><br>Minor criteria:<br>f) Abnormal ECG findings: ventricular arrhythmias (NSVT, multifocal PVCs), bundle branch block, axis deviation, or abnormal Q waves.<br>g) Perfusion defects on SPECT.<br>h) EMB: monocyte infiltration and myocardial interstitial fibrosis.

manifestation(s) have been reasonably excluded.		
*In general, 'probable involvement' is considered adequate to establish a clinical diagnosis of CS.<br>**1) Bilateral hilar lymphadenopathy; 2) elevated serum ACE levels or serum lysozyme levels; 3) elevated serum sIL-2R levels; 4) abnormal accumulation in ^{67}Ga citrate scintigraphy or FDG-PET; and 5) a high percentage of lymphocytes with a CD4/CD8 ratio of >3.5 in BAL.		

ACE, angiotensin converting enzyme; BAL, bronchoalveolar lavage; CMR, cardiac magnetic resonance; CS, cardiac sarcoidosis; CT, computed tomography; ECG, electrocardiogram; EMB, endomyocardial biopsy; FDG, 18F-fluorodeoxyglucose; LVEF, left ventricular ejection fraction; NSVT, non-sustained ventricular tachycardia; PET, positron emission tomography; PVC, premature ventricular complexes; sIL-2R, soluble interleukin-2 receptor; SPECT, single-photon emission computed tomography; VF, ventricular fibrillation; VT, ventricular tachycardia

Supplemental Table 2. Characteristics of patients with negative FDG-PET and ^{67}Ga scintigraphy

	62-years-old, male	74-years-old, female	77-years-old, female
Diagnostic criteria fulfilled	Clinical diagnosis of JCS	Clinical diagnosis of JCS	Clinical diagnosis of HRS and JCS
Endomyocardial biopsy	+	-	+
Histology of the heart	-	NA	-
Histology of extra-cardiac organs	-	-	Lung
Clinical findings of extracardiac organs	Lung	Eye	Lung
High-grade AVB	-	-	-
sustained VT or VF	+	+	+
Abnormal ventricular wall anatomy	-	Septal wall thinning	-
LVEF	47%	15%	40%
Focal ventricular wall asynergy	Unknown	+, distribution is unknown	+, distribution is unknown
LGE in CMR	+, distribution is unknown	+, base-mid anteroseptal, septal, inferior. Apex.	+, base-mid anteroseptal, septal.

AVB, atrio-ventricular block; CMR, cardiac magnetic resonance; FDG, 18F-fluorodeoxyglucose; HRS, Heart Rhythm Society; JCS, Japanese Circulation Society; LGE, late gadolinium enhancement; LVEF, left ventricular ejection fraction; PET, positron emission tomography; VF, ventricular fibrillation; VT, ventricular tachycardia.
